# Supplementary material for: Are carbon emissions trading and green financial instruments synergistic? -Comprehensive quantitative research based on content analysis
Source: PLoS One. 2024 Mar 7;19(3):e0298601. doi: 10.1371/journal.pone.0298601 (PMC10919601; doi:10.1371/journal.pone.0298601)
Supplement: S1 Data — (ZIP) [file pone.0298601.s001.zip › Data/地级市Z_F工作报告.zip!/╜¡╬≈/─╧▓2╩╨/└┤╫╘╣1⁄2╓┌║┼í╛┬φ┐╦╩2╛▌═°í┐_2008.docx]

各位代表：

现在，我代表市人民政府，向大会作政府工作报告，请予审议，并请各位政协委员和列席会议的同志提出意见。

一、2007年工作简要回顾

过去的一年，在省委、省政府和市委的正确领导下，全市上下高举中国特色社会主义伟大旗帜，以邓小平理论和“三个代表”重要思想为指导，全面落实科学发展观，努力构建和谐社会，解放思想，善谋实干，紧紧围绕富民强市目标，全力做好“两篇文章”，打造“一个基地、三个中心、四个南昌”，圆满完成了市十三届人大二次会议确定的各项目标任务。

——在新的历史平台上，经济继续保持又好又快发展。主要经济指标保持两位数增长，预计地区生产总值1409亿元，增长15.5%；财政总收入190.6亿元，增长26.6%；地方一般预算收入87.2亿元，增长28%。投资、消费、出口齐头并进。预计城镇以上固定资产投资773.45亿元，增长30%；社会消费品零售总额427.22亿元，增长19.2%；南昌地区出口总额23亿美元，增长38.4%。经济结构更加优化，一、二、三产比例为5.8：55.1：39.1。单位生产总值能源消耗下降率控制在4%以上。“六大板块”各显特色，协调发展。

——城乡面貌进一步改观，“三城”创建初战告捷。全市重大重点工程稳步推进，城市基础进一步夯实，城市功能进一步完善；以“三城”创建为目标，大力开展城市管理年活动，国家园林城验收基本通过，接受了省级文明城市测评和全国文明城市2007版指标体系测试；社会主义新农村建设有新进展，涌现出了一批国家级、省级重点镇、示范镇和文明村镇。

——民生工程进展顺利，城乡群众得到新的实惠。民生工程八个方面顺利实施，53件实事件件落实，城乡群众共享改革发展成果。城镇居民年人均可支配收入13253元，增长17.9%；农民年人均纯收入4985元，增长13.5%。被《中国经济周刊》评为“2007中国十大和谐城市”之一。

回顾2007年政府工作，主要抓了以下五个方面：

——坚持大开放主战略和新型工业化核心战略，壮大了经济总量，提升了经济质量。突出表现在：开放型经济有新突破。预计实际利用外资12.3亿美元，增长17.1%；实际利用内资347亿元，增长12%；投资1000万美元以上的外资项目42个，投资5000万元以上内资项目145个；已有28家世界500强企业、25家国内200强企业投资南昌。加大服务外包产业扶持力度，建立服务外包人才实训基地和对日服务外包基地。工业实现第二个“三年翻番”。坚持以重大项目为突破口，“3010”工程进展顺利，已有晶湛科技二期、富昌科技、南钢铁钢系统技改、联志电子产业园4个投资30亿元以上项目，晶能光电硅衬底发光二极管、中兴软件、一元数码、神基电子、泰豪科技静音电源、江铃控股乘用车、江铃V348全顺车改造等18个投资10亿元以上项目开工建设。一批高新技术企业落户园区，一批国有企业顺利改制，名牌战略实施有了新起色，工业经济效益不断提升。预计全市规模以上工业增加值385亿元，增长23%；主营业务收入1205亿元，增长27%；实现利税124亿元，增长23%；工业增加值和主营业务收入在2004年基础上实现第二个“三年翻番”。农业生产稳中有升。认真贯彻支农惠农政策，全面实施粮食直补、良种补贴、综合补贴、农机具补贴，加大农业基础设施投入，加快实施农业标准化，积极改善农业生产条件，农业综合生产能力大幅提升。预计粮食总产215.07万吨，连续四年实现丰收，再创历史新高；畜禽、鱼、肉、蛋、奶产量持续增加。县区工业园依托优势、形成特色。蒋巷、黄马等现代农业示范园正在打造。市级以上农业产业化龙头企业达135家，军山湖清水大闸蟹获国家地理标志保护产品称号。现代服务业发展迅猛。制定了商贸物流发展规划和配套措施。洪城大市场改造、紫金城时尚生活中心、鸿顺德商贸城二期、恒茂国际都会、联发大厦和国际金融中心等商业项目建设进展顺利；华中国际工业原料物流、厦门海投物流等项目落户南昌。金融产业蓬勃兴旺，被评为中国最具投资价值金融生态城市；全市金融机构存款余额超过2000亿元、贷款余额超过1800亿元；中信银行、都帮财产保险、民生人寿保险等一批金融机构进驻南昌，南昌市商业银行顺利增资扩股。出台了扶持企业改制上市及上市公司再融资的政策，正邦科技顺利上市，全市上市公司已达17家；18家重点企业列入上市后备名单、进入辅导期，资本经济发展初显端倪。加快旅游业发展，实现旅游总收入64.4亿元，增长16.6%。成功举办第六届金秋经贸活动月、承办赣台会、第22届全国医疗器械展、第三届中国（江西）红色旅游博览会等大型会展活动，荣获2007年“中国节庆产业十大节庆城市”奖。全民创业势头强劲。预计非公有制经济实现增加值778亿元，增长17.5%，占全市GDP比重达55.2%。巴士在线与中央电视台等成为战略合作伙伴，为目前国内唯一拥有国家广电总局批准播出和传输资质的民营企业。

——坚持新型城镇化战略，城乡面貌明显改观。完成城市总体规划修编并上报，完成铁路西客站地区发展规划、旧城区控制性详细规划、朝阳洲地区控制性详细规划调整方案等，启动南昌大桥——生米大桥沿江建筑景观城市设计规划等重点规划。按照南昌中央商务区的规划，加快红谷滩新城建设，推进红角洲——“南昌水城”、金融大街、总部基地、创意产业园建设；西外环高速正式通车，完成火车站西广场改造、南昌长途客车站扩建工程，完成三店西路、青云谱路、火电路铁路下穿通道工程；完成红谷滩秋水广场至八一大桥段景观工程和赣江市民公园二期工程渔舟湾湿地公园建设；完成豫章路、西湖路精品街区改造；加快青山湖综合整治二期项目和五干渠综合治理；洪都大桥、英雄大桥主桥工程进展顺利；启动阳明路下穿青山湖隧道、高新南大道（三期）、青山湖综合整治（二期）工程；昌九城际铁路、向莆铁路开工建设；昌北国际机场扩建改造抓紧推进；新昌电厂隆重奠基；新建扩建及改造电网工程12项；麦园垃圾场填埋气发电工程并网发电，减少碳气排放并进入国际市场交易；加快燃气管网建设步伐，着手对接管输天然气进入我市前期准备工作。九江—南昌—樟树输油管道铺设完工。新开通公交线路6条，新增、更新公交车197辆，其中空调公交车107辆；改扩建双港水厂，提升了饮用水源水质；红谷滩污水处理厂一期和象湖污水处理厂建成并投入运营，全市日处理污水能力达到81万吨；加大玉带河改造、艾溪湖、抚河截污和红谷滩、象湖污水收集管网工程推进力度；开通南昌——上海海铁联运，出口加工区经国家验收并投入使用。深入开展城市管理年活动。创建国家园林城突出立体绿化，社区、单位绿化，道路绿化和建设工程项目绿化，中心城区新增绿地43.47万平方米，绿化覆盖率达40.2%，绿地率达37.9%，人均公园绿地8.4平方米；创建全国文明城以深入开展文明南昌行动为重点，大力弘扬“大气开放、诚信图强”的城市精神，强力主攻薄弱环节，不断提升现代文明；创建国家环保模范城启动“绿色生态南昌建设”六大专项行动，14项市级企业限期治理项目实施，医疗废物处置中心建成并投入使用，一批建成项目通过环保验收；全年空气质量优良率95.3%，连续四年列中部省会城市第一位；饮用水源水质达标率99%以上，水环境质量在全国继续名列前茅。

围绕繁荣农村经济、促进农民增收，增加财政投入，推进社会主义新农村基础设施建设。乡镇总体规划全部完成，782个行政村完成了村庄规划。按照让农民“走平坦路、喝干净水、上卫生厕、住整洁房、用洁净能源”的要求，突出抓好669个试点自然村的“三化（划）三改”整治建设。成功举办第七届全国村长论坛。实施40座水库除险达标建设，完成大中型机电泵站更新改造4.52万千瓦，解决11.47万农村群众饮水安全问题。农村植树造林、流通新网工程、自然村道路硬化、卫生院和敬老院改扩建等工作进一步推进，提升了农村社会公共服务能力。

——坚持科教兴市、人才强市战略，各项社会事业进一步发展。一是优先发展教育，全面落实义务教育经费保障机制，启动城区九年一贯制义务教育示范学校建设，率先在红谷滩新区实施“优质教育全覆盖”工程，职业教育进一步加强。改造中小学危房1.78万平方米，顺利通过国家“两基”达标验收。小学、初中入学率分别为100%和99.3%，升学率分别为100%和87.6%。二是立足人才强市，出台《关于高层次人才队伍建设的若干意见》，人才引进、培养、使用机制不断完善，人才队伍不断壮大，人才素质不断提高。三是坚持科技第一生产力，积极推进国家半导体照明工程产业化基地、国家科技兴贸出口创新基地、国家火炬计划软件基地金庐软件园、生物医药产业化基地、农业示范基地建设；做强做大软件产业，泰豪科技、先锋软件、思创数码进入全国软件行业100强。实施国家级创新基金项目16项、省级科技项目102项，成为全国知识产权示范创建城市，高新区被批准为国家知识产权试点园区。四是以建设“健康南昌”为目标，加强疾病预防控制，市疾控中心大楼完成主体建设，江西南昌紧急救援调度指挥中心正式启用；新建和改扩建572所村卫生室、63所乡镇卫生院，建成272个社区卫生服务机构。启动红谷滩新区“优质卫生保健服务全覆盖工程”，打造“十五分钟医疗服务圈”。五是推动文化大发展大繁荣，隆重纪念建军八十周年，开展了十大庆祝活动，八一起义纪念馆新陈列馆开馆，成功举办第二届中国南昌军乐节。加快江西艺术中心建设，启动有线电视数字平移工作，广播电视覆盖率分别达到98.77%和97.32%。六是围绕“打体育牌、唱崛起戏”，成功获得第七届全国城市运动会承办权，在第六届城运会上，获金牌5枚、银牌1枚、铜牌5枚，促进了群众性体育活动的开展。

——坚持可持续发展战略，促进人与社会、自然和谐相处。坚持共建共享，扎实推进民生工程。全年省级以上新增财政投入4.45亿元，市本级新增财政投入3.5亿元用于民生工程。全年新增城镇就业8万人，下岗失业人员再就业3.1万人，安置“4050”困难人员1.17万人，新增转移农村劳动力3.5万人，跨省劳务输出23.8万人。统筹范围内的离退休人员养老金100%按时足额发放，依照国家和省政策对符合条件的人员进行了补助和调资；城乡低保居民应保尽保，调高了城乡低保补助标准。扎实开展价格监测监管工作，居民消费价格总水平低于全省、全国平均水平；积极应对和疏导部分物价上涨矛盾，为城乡低保对象和五保户先后发放一次性肉价补贴和其他物价补贴。对公办义务教育阶段学生55.62万人全部免除学杂费，对义务教育阶段13.5万困难学生免除教科书费，发放高中和中职贫困生助学金，补贴义务教育阶段寄宿生生活费，对考入大学的困难家庭学生一次性补助。城镇职工医疗保险覆盖面不断扩大，参保人数达51万人，完成全年任务120%，参保率60%以上；积极推进农村新型合作医疗，参合农民达195.4万人，参合率89.84%；在东湖区试点基础上，全面启动城镇居民基本医疗保险，参保人数51.14万人，完成全年任务100%；大力实施城乡低保家庭医疗救助，率先在全国开展社区卫生服务与城镇居民基本医疗保险对接。采取发放租金补贴为主、实物配租和租金核减为辅的形式，共有2046户低保家庭享受廉租住房政策；在城南、城东、红谷滩、昌北共规划建设了四个经济适用房住宅小区共70万平方米，公开摇号、阳光操作，可解决近万户中低收入家庭的住房困难。积极推进低洼危旧房、城中村改造。

坚持既要金山银山、更要绿水青山，全面落实人口、资源、环境各项政策。严格执行计划生育政策，完成了省下达的计生目标任务，计生奖励优惠政策惠及20万余户群众，综合治理性别比工作初见成效。严格执行国家节能降耗、环境保护政策，坚持“上大压小”，严厉打击非法排污企业；加强植树造林和水土保持工作；以省委、省政府“国土资源管理年”活动为契机，坚决贯彻落实基本农田保护“五不准”和耕地保护责任制；积极推进造地增粮富民工程，确保了全市耕地占补平衡；进一步完善建设用地审批程序和公开出让制度，依法妥善处理土地历史遗留问题，大力推行集约节约用地，提高土地利用效率。

以创建和谐平安南昌为目标，切实做好稳定工作，维护了社会良好秩序。积极开展打黑除恶、命案侦破、打击“两抢一盗”以及反盗窃等专项行动，对重点工程、重点企业、公共聚集场所、校园周边环境、水上治安秩序等进行专项整治或集中整顿；深入推进公安“三基”（抓基层、打基础、苦练基本功）工程建设和信息化建设，完善了治安防控体系，严厉打击各类违法犯罪行为。认真抓好生产安全、交通安全、消防安全、森林防火安全、产品质量安全和食品卫生安全。建立健全分类管理、分级负责、条块结合、属地为主的应急管理体制，有效应对各类自然灾害、公共事件等。认真做好信访接待工作，千方百计化解矛盾。狠抓“两所一庭”建设，积极开展法律援助。全市没有发生大规模群体事件、严重暴力性事件、重大治安事件、恶性火灾事件和重特大生产经营性事故，交通事故四项指数全面下降，继续成为全省和谐稳定的“首善之区”。

——坚持依法行政，建设法治效能政府。提请市人大常委会审议地方性法规2件，制定政府规章6件；清理现行政府规章，废止规章4件，修改规章3件；审结行政复议案件142件，维护了公民、法人和其他组织的合法权益。进一步深化行政审批制度改革，认真执行《行政许可法》，推行行政许可“两个集中”，简化审批程序；扎实开展普法和依法治市工作，大力推行行政执法责任制；以开展“整顿‘五风’，提高能力，促进发展”和“政风行风评议评价”活动为契机，加大纠风治乱和效能监察力度，进一步规范了行政行为。政府各部门、各执法单位能够文明、公正执行公务，取得明显成效。市政府被授予全国政务公开先进单位和全省依法行政示范单位。进一步推行电子政务，完善“中国南昌”门户网站体系，完成“数字南昌”二期工程建设，提高社会管理和公共服务水平。坚持科学民主决策，广泛听取社会各方面的意见和建议，自觉接受人大及其常委会的依法监督、政协的民主监督和群众监督、舆论监督。

与此同时，大力开展国防教育，加强人防建设，推进优抚安置和双拥工作，荣获了全国、全省双拥模范城“六连冠”；外事侨务工作成绩显著，新缔结国外友好城市两对；民族宗教、对台事务工作继续加强，统计、审计、物资、参事、档案、地方志、保密、老龄、妇女儿童、慈善、残疾人、消费者权益保护、防震减灾等工作，都取得新的成绩。

各位代表，2007年，南昌经济社会继续保持又好又快发展，来之不易。这是全市人民在市委正确领导下，认真贯彻党中央、国务院和省委、省政府大政方针，团结奋斗、扎实工作的结果；是市人大依法监督和市政协民主监督的结果；是市各民主党派、工商联、无党派人士、各人民团体和驻昌单位、人民解放军、武警官兵以及离退休老同志共同努力和大力支持的结果。在此，我谨代表市人民政府，向全市人民，向所有关心、支持南昌发展的同志们、朋友们，表示衷心的感谢并致以崇高的敬意！

在总结2007年工作的同时，我们也清醒地认识到，与中部其他省会城市相比，我市经济总量还不大，城市综合实力还不强，仍属欠发达地区。主要是：高科技、有竞争力的企业和产业集群还不多；城乡管理水平有待进一步提升；群众增收和生活质量提高的压力还不小；资源节约和环境保护任务较重；政府效能仍需继续提高。对此，我们将采取切实措施，认真加以解决。

各位代表，当前和今后一个时期，南昌正处在一个重要的发展阶段，机遇和挑战并存。随着国际产业分工纵深发展和经济全球化趋势进一步加剧，国与国之间的合作与交流更加密切，和平与发展仍然是时代主题；我国经济继续在高位快速健康运行，中部崛起战略的实施和深入推进，东南沿海地区产业进一步向中西部转移，中部在全国经济大发展中发挥着越来越重要的作用。南昌作为京九线上唯一的省会城市和唯一与“长三角”、“珠三角”、闽东南三角区毗邻的省会城市，有着得天独厚的区位优势、资源优势和低成本优势；随着近年来打造“一个基地、三个中心、四个南昌”步伐的加快，产业优势开始显现，正处于加快崛起的重要机遇期。但是，全球经济发展速度减缓，原材料价格上涨幅度较快，国际经济环境中的不确定因素增多；国家加大宏观调控力度，实施从紧的货币政策和稳健的财政政策，防止经济增长由偏快转为过热，防止价格结构性上涨演变为明显通货膨胀，要素制约因素加大，发展平台更高，这都要求我们在工作中积极应对，趋利避害，正确把握。

二、2008年工作目标和重点工作

2008年，是全面贯彻落实党的十七大精神的第一年，也是加快南昌崛起的重要之年。贯彻落实党的十七大精神，加快富民强市步伐，促进南昌快速崛起，是历史赋予我们光荣而又神圣的使命。今年政府工作总的要求是：高举中国特色社会主义伟大旗帜，全面落实科学发展观，着力转变经济发展方式，努力构建和谐社会，紧紧围绕富民强市目标，深入做好“两篇文章”、打造“一个基地、三个中心、四个南昌”和“六个走在全省前列”的总体要求，以“创业富民、创新发展”为主线，以重大项目为突破口，坚持发展第一要务、环境第一品牌、民生第一基石、稳定第一责任，团结拼搏，排难而进，在更高层次上推动南昌经济社会又好又快发展。

综合考虑各种因素，今年全市国民经济和社会发展的主要预期目标是：

地区生产总值增长14.5%；

财政总收入增长16%；

地方一般预算收入同口径增长16.3%；

全社会固定资产投资增长22%；

社会消费品零售总额增长16.5%；

实际利用外资增长10%；

实际利用内资增长10%；

城市居民年人均可支配收入增长9%以上；

农民年人均纯收入增长9%以上；

单位生产总值能源消耗比上年下降4%；

人口自然增长率控制在8.15‰以内；

居民消费价格总水平控制在上年涨幅以内。

为了实现今年工作总的要求和主要预期目标，必须重点抓好以下四个方面的工作：

（一）坚持发展第一要务，突出项目推进，不断提高南昌经济发展的集聚力和辐射力

——坚定不移地实施大开放主战略，不断提高开放型经济发展水平。紧紧围绕做大财政蛋糕、增加劳动就业，进一步提高招商引资水平。大力创新招商引资方式，依托南昌区位、资源及环境优势，注重招商选资和招强引大，着力引进世界500强和国内200强企业，引进高新技术和延伸完善产业链的配套企业，充分发挥引资在拉动自主创新、产业升级、区域发展中的积极作用。积极参加省政府组织的招商推介活动，做好项目承接；精心组织全市重大招商引资活动，办好第七届金秋经贸活动月，继续开展招商万里行活动，派遣小分队开展针对性招商，创新定向招商、代理招商等模式。进一步加大出口对经济发展的拉动作用，大力发展外经外贸。扶持南昌出口加工区加快发展，重点引进带动作用大、辐射功能强、示范效应高的项目入驻。努力扩大机电产品、高新技术产品出口份额。加快建立光机电一体化、电子信息、汽车、服装等出口产业基地。创新对外投资和合作方式，扩大对外工程承包和劳务输出。支持软件和服务外包产业做强做大，积极申报中国服务外包示范区和中国服务外包培训中心，加强与微软、IBM公司的战略合作，力争引进新的知名企业入驻，促进服务外包产业的集聚。

——坚持改革攻坚战略，不断创新机制体制。继续深化和推进国有企业改革，积极探索国有及民营企业资产经营管理方式，围绕提高资源集约利用效率，深化要素配置市场化改革，鼓励更多的资产资本向企业集团集聚，促进企业裂变扩张；推动优势企业上市融资、已上市公司增资扩投或整体上市，加快商品经济形态向资本经济形态转变。进一步完善商业用地净地出让制度和工业用地招拍挂制度，积极探索建立污染物排放总量控制制度、排污权交易制度等，逐步建立能源、资源、排污权使用差别化价格、阶梯式价格调节机制；进一步改革公共服务供给模式，探索政府购买公共服务的有效方式，促进公共服务主体多元化。推动公用行业市场化改革和社会化运作，深化城市基础设施建设投融资体制改革，盘活城市资产，创新融资方式。

——坚持科技第一生产力，努力提高城市核心竞争力。以建设创新型城市为目标，着力突破制约经济社会发展的关键技术。加强与高等院校、科研院所的合作，促进科技成果转化。完善相关扶持政策，大力采用高新技术改造提升传统产业，大力发展高新技术产业，推动高新技术产业化和产业高新技术化。加强创新载体建设，积极启动瑶湖科技城建设，加快农业科技示范园建设，完善农业科技服务体系，建立健全知识产权管理体系、服务体系、保护体系。

——以大力实施“3010”工程为重点，加快先进制造业重要基地建设步伐。强化工业经济主导地位，依托高新、昌北两个国家级开发区等重点园区，发挥英雄、桑海等开发区各自特色，积极引导工业项目向开发区、工业园区集中，支持各开发区差异发展、扬优成势。大力支持电子信息和家电、汽车制造和配套、医药和食品、纺织服装、新材料等五大支柱产业发展。策应全省“十百千亿”支柱产业工程，继续大力推进工业“3010”工程，对科技含量高、投入产出比大、经济带动力强的项目在土地、资金等方面给予重点倾斜，坚定不移地走高科技、低消耗、低污染、高效益的新型工业化路子。倾力支持中央和省驻昌企业做强做大，大力扶持国资公司等市属骨干企业改革发展，支持引资入园企业达产增效，鼓励民营企业裂变扩张，进一步提升我市内源型经济活力。注重节能减排工作，开展清洁生产，发展循环经济，大力实施名牌战略，提升标准化水平，进一步提高工业经济效益。

——大力发展现代农业，做强做大县域经济。贯彻“工业反哺农业、城市支持农村”和“多予、少取、放活”方针，落实各项支农、惠农政策，突出现代农业、县域经济、新农村建设三个重点，促进农业增效、农民增收、农村和谐、城乡协调。财政支农投入的增量要明显高于去年，固定资产投资用于农村的增量要明显高于去年，政府土地出让收入用于农村建设的增量要明显高于去年。继续抓好优质粮食产业工程，稳定发展粮食生产。大力发展畜牧水产生产，加强蔬菜基地建设，突出抓好近郊蔬菜基地向中远郊梯度转移。加快推进畜牧体制改革，加强动植物疫病防控，尽快建立健全农产品质量安全监管长效机制，提高农产品质量安全水平。通过实施“一村一品”、“双带”致富工程等，因地制宜发展农村经济；大力发展农民专业合作组织，稳定和加强农业基层服务体系；加大农民工培训力度，培育有文化、懂技术、会经营的新型农民，多渠道转移农民就业。大力推进农田水利基本建设，重点实施水库除险、泵站改造、渠系整治等工程，加大以改造中低产田和建设标准农田为主要内容的农业综合开发力度，提高农业综合生产能力。认真组织实施省政府农业产业化“双十双百双千”工程，精心运作市农业产业化信用担保公司，引导扶持龙头企业上规模、上档次、增效益。做强县域经济实力，推动小蓝开发区、昌东工业园、昌南工业园和新建、进贤、安义工业园（区）加快发展，推动蒋巷、黄马等现代农业科技示范园形成特色。

——以打造区域性商贸、物流、金融中心为重点，加快发展现代服务业。优化商贸业布局。稳步提升老城区市级商业中心水平，加快推进红谷滩中央商务区和重点商贸服务设施建设。支持城东、城南、城西、城北区域商业中心的规划建设。巩固完善社区商业网点和农村“万村千乡”商业网点。整合物流业资源。突出与口岸、专业市场、产业园区相配套的物流设施建设，加快推进华中国际工业原料物流、昌北航空物流、向塘铁路物流等物流中心建设。提高金融业集聚能力。支持在昌金融机构扩大业务、防范风险；力争年内再引进1家外资银行、1家股份制银行入驻南昌；加快南昌市商业银行上市步伐，推进洪都农村信用联社改制；积极组建安义村镇银行。推动金融进社区试点工作，推进红谷滩金融大街建设；完善银企对接服务机制，鼓励银行对成长性经济提供更多的信贷支持；加大企业上市扶持力度，重点扶持巴士在线、晶能光电、恒大高新、泰豪软件、汇仁药业、煌上煌等企业，力争实现2—3家企业上市。打响旅游会展业品牌。突出红色摇篮、绿色家园主题，挖掘南昌文化底蕴，加快制定旅游发展规划；加大景区开发力度，提升景区创A水平，推进“八大山人”风景区和文化产业园建设，完成南昌中国傩园建设。加强区域性国际性旅游合作与交流，强化城市旅游形象的宣传促销。大力发展会展经济，积极申办中国食品博览会、全国农机交易会、全国糖酒交易会等全国性大型会展，利用长三角城市会展联盟平台，积极引进2010年上海“世博会”1—2个活动到南昌举办，努力打造培育1—2个南昌品牌展会。

——着力推进全民创业，加快非公有制经济发展。坚持以创业促就业、以创业促发展方针，完善创业服务体系，激发创业潜能，使更多的劳动者成为创业者。采取整体规划、滚动发展的方式，加快建设步伐，力争在全市打造出20个设施配套、功能齐全、服务高效的中小企业创业基地。坚持政府扶持、市场运作相结合，以融资担保、信用服务、人才培训、信息网络、技术支持和法律政策为主要内容，逐步建立起中小企业综合服务体系。大力实施中小企业成长工程，积极扶持中小企业快速发展壮大。

（二）坚持环境第一品牌，规划、建设、管理并重，不断提升南昌的软硬实力

以承办第七届全国城市运动会为总抓手，坚持高起点规划、高标准建设、高水平管理，进一步提升承载功能和城市品位。重点组织编制城运会场馆布局规划、城市基础设施和配套设施规划、城东和城北地区控制性详规、城市重要道路过街设施规划等。加强国土资源管理，启动第二次国土资源调查，完成土地利用总体规划修编，把节约集约用地作为转变经济发展方式、优化产业结构的突破口，严格落实土地管理和耕地保护责任制，保护基本农田；切实按照国家产业政策和用地政策，严格执行工业用地、经营性用地、矿业权招标拍卖挂牌出让制度。整顿和规范矿产资源开发秩序，健全矿产资源有偿占用制度和矿山环境恢复检查机制。

继续推进“一江两岸”工程，切实加快以交通、能源、水利等为重点的工程项目建设。一是交通工程。继续推进洪都大桥、英雄大桥工程建设，阳明东路下穿青山湖隧道工程、高新南大道（三期）工程、铁路西客站工程，青山湖南大道南延、广州路东延工程，昌北国际机场扩建，向莆铁路、昌九城际铁路、昌南大道等重大重点项目建设；抓好紫阳大道、昌东大道、解放东路等改造工程，以及北京东路跨艾溪湖连接紫阳大道大桥工程，积极争取城市轨道交通项目国家立项。二是能源工程。加快新昌电厂建设步伐，增强配电网整体供电能力。加快城市燃气管网建设，积极引进天然气，提高管网覆盖率和燃气的气化率。三是供水和水利工程。抓好城区供水水源及管网工程，启动建立供水管网信息调控保障系统和城市应急水源规划建设，提高城市供水能力和质量。推进朝阳电排站工程和乌沙河整治工程。四是朝阳地区开发建设工程。全面启动朝阳地区路网、水网、管网建设。五是场馆建设工程。抓紧南昌国际体育中心、射击中心、水上运动中心和相关接待设施建设。

以创建全国文明城、国家环保模范城为目标，切实加强城乡管理。一是加大城市规划的执行力度，加强城市管理机构建设，组织协调督促各级各部门切实抓好城市管理，建立健全条块结合、责权利统一的城市管理长效机制。二是严格按照新的全国文明城市测评体系标准，积极开展全国文明城市活动年、六进社区、诚信南昌、为百姓办百件事等四项活动，责任到人，落实到位，按要求达标。三是继续推进“畅通工程”，采取综合手段，努力缓解交通拥堵。着力完善老城区快速交通干道系统，改善次干道和支路系统，科学组织交通微循环，提高现有道路通行能力；改善路容路貌，加快道路绿化，打造“畅、洁、绿、美、安”的公路交通环境；实施“公交优先”战略，完善城市公共交通基础设施，鼓励社会力量建设停车场，开展疏堵治乱降事故工作。四是加强生态环境保护，规划和启动建设昌南生活垃圾焚烧发电厂，推进垃圾减量化、资源化改革的实施，加强植树造林和水土保持工作；深入开展“创建国家环保模范城”活动和“绿色生态南昌建设”专项行动，全面推进城市第一次全国污染源普查工作，完成环境监察大楼及污染源在线监控系统建设；逐步完善城区污水收集管网系统，完成玉带河南支、东支、桃花龙河截污工程，启动黄家湖截污工程，加快老城区雨污分流步伐，新城区一律采用雨污分流制；进一步打击非法排污企业，加快困难企业居民生活区卫生环境整治；五是推进城市管理标准化建设，组织制定实施城市公共事业建设、管理相关标准，逐步与国际化接轨。六是着力抓好6个改革与发展试点镇、10个示范镇、14个省级重点镇建设，继续推进乡镇集镇环境卫生、镇容镇貌整治和长效管理。七是突出抓好以试点村整治和示范区建设为主要内容的新农村建设试点工作。启动“农村小康环保行动计划试点市”工作；抓好700个左右试点自然村整治建设。突出农村村庄规划、生态绿化、环境美化，改水、改厕、改路，继续推进农村植树造林、自然村道路硬化、中小学危房改造、卫生院的改扩建等基本建设，提升农村社会事业发展水平和公共服务能力。

在抓好城市基础硬环境的同时，继续优化投资发展软环境，进一步打响“四最”品牌。简化办事程序，完善“一站式”集中联合办公模式和网上办事服务，推进行政审批“两个集中”。大力开展优化投资环境主题年活动，继续营造亲商、安商、富商的浓厚氛围。建立和完善与国际惯例和沿海城市对接的政策法规体系，营造开放的制度优势；加强国际间友好城市的协作与交流，加强国内区域合作，实现市场、设施、生态对接，营造开放的协作优势；加强海铁联运，加快保税物流中心建设，完善出口加工区服务，营造开放的成本优势；加快电子口岸建设，加强行政服务中心和招投标中心等政务平台建设，营造开放的服务优势。

（三）坚持民生第一基石，突出统筹兼顾，不断提高社会的进步程度

加快推进以改善民生为重点的社会建设。一是优先发展教育，抓好市区基础教育网点布局调整的详细规划编制工作，加大城区九年一贯制义务教育示范学校建设力度，新增教育经费重点支持农村和薄弱学校的建设。调整中等职业学校布局和结构，推动高等职业教育发展。二是全面实施人才强市战略，贯彻落实《关于进一步加强高层次人才工作的若干意见》，加大各类人才尤其是高层次创新人才、优秀企业家、高级技能人才的培养和引进力度，优化人才成长环境。三是以建设“健康南昌”为目标，合理布局卫生资源，增设新区医疗卫生服务网点；加强疾病预防控制，加强市血站能力建设；加快农村卫生和社区卫生发展，逐步建立“分级保障、低水平、全覆盖、大病救助、特殊人群救助”的全民医疗保障体系；大力开展爱国卫生运动。四是着眼建设文化大市、文化强市，加快文化事业发展。编制公共文化设施布局规划，加强公共文化基础设施建设。实施新四军军部旧址陈列馆、八大山人纪念馆改扩建工程，加快报业大厦建设。继续深入开展“扫黄打非”工作和“反盗版天天行动”，举办“和谐南昌”系列文艺展演活动，营造健康向上、文明和谐的文化环境。五是按照国家“三网融合”的要求，完成有线电视数字平移工作，加快有线电视网络双向改造，为用户提供多功能信息咨讯服务。六是扎实推进第七届全国城市运动会的各项筹备工作，积极开展群众性体育活动，迎接北京奥运会的召开。

坚持“在共建中共享，在共享中共建”，认真落实国家和省有关政策，切实把关注民生作为构建和谐社会的首要任务来抓，以人为本，从人民群众最关心、最直接、最现实的利益问题入手，新增财力重点向困难群体倾斜、向农村倾斜、向基层倾斜、向社会事业倾斜。今年民生工程突出八个方面、办好66件实事。

——就业再就业方面。一是通过发展开放型经济、城市产业、个私民营经济、非正规就业组织和劳务输出，力争新增城镇就业8万人，使城镇就业率达到94%以上。二是通过增加社会公益岗位、加强技能培训等，重点做好“零就业家庭”和“4050”人员的就业工作。力争安置“4050”从员就业4500人，“零就业家庭”安置率100%。三是突出抓好工业园区劳动力的对接、跨省劳务输出的组织引导、维护进城农民工合法权益、农民工职业培训等工作，力争新增转移农村劳动力3万人。

——强化生活保障方面。一是做好养老保险工作。国家和省出台的企业职工生活保障政策确保到位；提高企业职工养老保险标准；在青云谱区、青山湖区、红谷滩新区试点的基础上，积极稳妥地推进失地农民养老保险。二是完善社会救助体系。扩大农村低保范围，适当提高农村居民最低生活保障补差水平和城市低保补差水平；继续改建、扩建、新建农村敬老院、福利院，进一步提高农村五保对象集中供养率；建立临时性救助制度，把低保边缘户和因天灾人祸引起生活困难的家庭纳入到救助范围；大力发展慈善事业，改善孤残儿童的生存、生活条件。三是抓好住房保障工作。设立廉租住房保障专项补助资金；抓好低收入家庭住房保障现状调查，抓紧制定城市低收入家庭资格认定办法；继续抓好经济适用住房建设和低洼危旧房改造；逐步扩大廉租住房覆盖面。四是切实抓好物价监管工作，确保市场和价格基本稳定。

——卫生医疗方面。将农民全部纳入新型农村合作医疗范围并提高补助标准；建立健全城乡医疗救助制度，提高医疗救助水平；将城镇居民全部纳入城镇居民基本医疗保险范围；扩大计划免疫规划范围，确保适龄儿童享受一类疫苗免费接种政策；如期实现全市血吸虫病疫情控制目标；加强农村药品“两网”建设及药品监测检验建设，提升食品药品检验能力；稳定低生育水平，提高出生人口综合素质；改善村卫生所、乡镇卫生院条件，培训医务人员；努力实现城市社区公共卫生和基本医疗服务全覆盖。

——济困助学方面。提前一年达到中央要求的城乡义务教育阶段学校生均公用经费标准，增加公办高校生人均经费；增加中职、技校生人均经费；城乡义务教育阶段学生全部免除学杂费、教科书费；对城乡义务教育贫困寄宿生生活费给予适当补助；对民办学校义务教育阶段学生学杂费进行补助；严格执行公办高中招收择校生的“三限”政策，严格规范各级各类学校收费行为；健全高中、中职和考取大学的特殊困难家庭学生资助政策体系；增加职业教育经费投入；对盲聋哑等特殊教育学校给予补助；切实做好符合条件的农民工子女义务教育工作，不得加收借读费等不合理费用；采取奖励方式支持各地化解农村“普九”义务教育债务；推进大中专院校放心粮油连锁配送中心建设。

——农村公益事业和基层组织建设方面。一是推进农村公益事业和水利设施建设。完成40座水库除险达标建设；实施5座大型机电泵站更新改造；加强1—5万亩圩堤整治，继续实施鄱阳湖治理第五、第六期建设；进一步解决农村人口饮水安全问题；继续兴建山塘门塘。二是进一步加大农村扶贫开发工作力度。抓好70个扶贫开发工作重点村的整村推进；鼓励重点村发展主导产业，农业产业化龙头企业到贫困地区建立原料和加工基地；继续抓好机关单位定点扶贫和光彩事业、“百企帮百村”等社会化扶贫。三是加大推进新农村现代流通服务体系建设力度。打造农村农资、日用品、农产品及再生资源等流通四大网络；构筑专业合作社、专业市场、专业协会等服务平台。四是推进基层民主政治建设。进一步加强村民自治、依法开展全市第七届村（居）民委员会换届选举工作；完善村务公开民主管理制度。

——生态建设和环境保护方面。抓好退耕还林和血防林建设、加快农村公路绿化、治理水土流失和保护生态公益林等工作；提高纳入补偿范围的公益林补偿标准，对森林资源保护工作先进县进行奖励；对市县淘汰落后产能进行奖励；支持各地建立节能监管体系和企业开展节能技术改造。

——社会管理和公共服务体系方面。建设数字城管系统；建设“南昌之窗——公众信息服务平台”；推进城市一卡通系统；完善街道社区管理体制改革，改善基层社区工作条件；积极开展“建设和谐社区示范单位”创建活动；增加市图书馆藏书；设立基层文物保护和博物馆维修专项经费，对县级文化馆、图书馆维修进行补助；支持基层文艺创作与繁荣工程建设；支持文化信息资源共享工程建设；开展全民读书工程和“农家书屋”工程建设；支持基层群众体育健身场所建设。

——公共安全方面。进一步完善社会治安防控体系，建设覆盖城区的视频监控系统；改善和加强城乡社区（驻村）警务工作，进一步加强“两所一庭”建设；完成南昌市战时人口疏散基地建设，推进重要经济目标的防护工作。

（四）坚持稳定第一责任，突出依法行政，努力打造为民、创新、务实、廉洁的政府

切实维护社会稳定。全面实施“五五”普法和依法治市规划，着力加强社会治安综合治理工作，继续深化公安“三基”工程建设和信息化建设，加强流动人口服务和管理，依法严厉打击刑事犯罪行为。切实抓好安全生产、交通安全和城市消防、森林防火工作，加强质量技术监督、市场监管和食品药品安全整治。加强突发事件的应急处置，提高危机管理和抗风险能力。加强法律援助工作，依法保护公民的合法权益；高度重视信访工作，完善信访制度，落实信访责任，加大矛盾排查、纠纷调处力度，切实解决征地、拆迁、改制等遗留问题。确保不发生重大群体事件、严重暴力事件、重大治安事件、恶性火灾事件和重特大生产经营性事故。

自觉坚持依法行政。进一步加强政府立法工作，推行行政执法责任制，推进综合执法改革，强化行政执法监督。自觉接受人大及其常委会的依法监督和政协的民主监督，自觉接受群众监督和舆论监督。认真办理人大代表议案、建议和政协委员提案，重视和发挥各民主党派、工商联、无党派人士和群众团体的作用。推行政务公开，进一步完善公众参与、专家论证和政府决策相结合的科学民主决策机制。

着力转变政府职能。按照权责统一、分工合理、决策科学、执行顺畅、监督有力的要求，深化行政管理体制改革，规范行政管理行为；进一步强化“立党为公、执政为民”理念，同心同德，善谋实干；加强公务员队伍建设，进一步提高对上级方针政策的理解力、执行力和操作力，自觉做到“两个求真务实”：一是求过程之真，务效果之实。说实话，干实事，求实效。二是求发展之真，务惠民之实。把精力放在谋发展上，把功夫下在为民、惠民上。

大力推进反腐倡廉建设。坚持标本兼治、综合治理、惩防并举、注重预防的方针，进一步建立健全惩治和预防腐败体系。严格执行领导干部廉洁从政各项规定，进一步落实党风廉政建设责任制，筑牢思想道德防线，坚决纠正损害群众利益的不正之风为重点，依法查处违法违纪案件。进一步转变干部作风，提高行政效能，不断优化经济发展环境。

各位代表，过去的一年是南昌取得骄人业绩的一年；新的一年，我们肩负着加快富民强市、构建和谐南昌、实现“六个走在全省前列”的重任。让我们更加紧密地团结在以胡锦涛同志为总书记的党中央周围，高举中国特色社会主义伟大旗帜，全面落实科学发展观，在省委、省政府和市委的正确领导下，大力弘扬“大气开放、诚信图强”的城市精神，以更加开阔的视野，更加有力的举措，更加务实的作风，团结一心，锐意进取，为实现经济社会又好又快发展、促进南昌在新的起点上快速崛起而不懈奋斗！
